# Paclitaxel effects on axonal localization and vesicular trafficking of Na_V_1.8

**DOI:** 10.3389/fnmol.2023.1130123

**Published:** 2023-02-13

**Authors:** Christopher A. Baker, Sidharth Tyagi, Grant P. Higerd-Rusli, Shujun Liu, Peng Zhao, Fadia B. Dib-Hajj, Stephen G. Waxman, Sulayman D. Dib-Hajj

**Affiliations:** ^1^Department of Neurology, Yale University, New Haven, CT, United States; ^2^Center for Neuroscience and Regeneration Research, Yale University, New Haven, CT, United States; ^3^Rehabilitation Research Center, Veterans Affairs Connecticut Healthcare System, West Haven, CT, United States; ^4^MD/PhD Program, Yale University, New Haven, CT, United States

**Keywords:** paclitaxel, chemotherapy, neuropathy, pain, sodium channels

## Abstract

Patients treated with paclitaxel (PTX) or other antineoplastic agents can experience chemotherapy-induced peripheral neuropathy (CIPN), a debilitating side effect characterized by numbness and pain. PTX interferes with microtubule-based transport, which inhibits tumor growth *via* cell cycle arrest but can also affect other cellular functions including trafficking of ion channels critical to transduction of stimuli by sensory neurons of the dorsal root ganglia (DRG). We examined the effects of PTX on voltage-gated sodium channel Na_V_1.8, which is preferentially expressed in DRG neurons, using a microfluidic chamber culture system and chemigenetic labeling to observe anterograde channel transport to the endings of DRG axons in real time. PTX treatment increased the numbers of Na_V_1.8-containing vesicles traversing the axons. Vesicles in PTX-treated cells exhibited greater average velocity, along with shorter and less frequent pauses along their trajectories. These events were paralleled by greater surface accumulation of Na_V_1.8 channels at the distal ends of DRG axons. These results were consistent with observations that Na_V_1.8 is trafficked in the same vesicles containing Na_V_1.7 channels, which are also involved in pain syndromes in humans and are similarly affected by PTX treatment. However, unlike Na_v_1.7, we did not detect increased Na_V_1.8 current density measured at the neuronal soma, suggesting a differential effect of PTX on trafficking of Na_V_1.8 in soma versus axonal compartments. Therapeutic targeting of axonal vesicular traffic would affect both Na_v_1.7 and Na_v_1.8 channels and increase the possibilities of alleviating pain associated with CIPN.

## Introduction

Chemotherapy-induced peripheral neuropathy (CIPN) is a debilitating side-effect of treatment with antineoplastic agents (Flatters et al., 2017; Staff et al., 2017; Ibrahim and Ehrlich, 2019). Current analgesics do not adequately address pain that frequently accompanies CIPN. Paclitaxel (PTX) is a prototypic chemotherapeutic used for a variety of cancers and exerts its antineoplastic effects through stabilization of polymerized microtubules, which impairs movement of macromolecular complexes during mitosis, thereby inducing cell cycle arrest (Gornstein and Schwarz, 2014). In highly specialized and polarized cells such as neurons, however, microtubules are also crucial for conveyance of molecules between different cellular compartments, especially along the length of axons. The neurons of the dorsal root ganglion (DRG) that convey tactile and nociceptive information from the periphery, for example, rely on microtubule-based motors to deliver ion channels from sites of synthesis in the neuronal cell body to axonal endings, which can be a meter or more away from soma (Akin et al., 2019). Because distal axonal regions are the sites of action potential electrogenesis in peripheral nociceptors, interference with such transport and associated inflammatory response to the chemotherapy would be likely to compromise neuronal function and contribute to the painful syndromes that are a side effect of PTX therapy (Staff et al., 2019).

PTX treatment has been shown to lead to hyperexcitability of DRG neurons (Li et al., 2018), which is accompanied by increased mRNA expression of voltage-gated sodium channels such as Na_V_1.7 (Xiao et al., 2016; Li et al., 2018; Akin et al., 2021). Na_V_1.7 is a determinant of the threshold for action potential firing (Vasylyev et al., 2014; Alexandrou et al., 2016), and genetic loss or gain of function in Na_V_1.7 are linked to dramatic alterations in pain sensitivity in humans (Bennett et al., 2019; Dib-Hajj and Waxman, 2019). Transport of Na_V_1.7 to DRG distal axonal endings in culture is enhanced by proinflammatory molecules and by PTX itself (Akin et al., 2019, 2021). In DRG neurons Na_V_1.7 co-traffics in vesicles with the related voltage-gated sodium channel Na_V_1.8 (Higerd-Rusli et al., 2022), which is also associated with human pain sensitivity syndromes (Bennett et al., 2019; Dib-Hajj and Waxman, 2019). Na_V_1.8 produces most of the current underneath the upstroke of action potential in DRG neurons and underlies their ability to sustain repetitive firing (Renganathan et al., 2001; Blair and Bean, 2002; Han et al., 2015), but its contribution to CIPN is unknown and any PTX-induced changes in its transport have yet to be demonstrated.

In the present study we examined the effect of PTX on Na_V_1.8 by leveraging our recently developed Optical Pulse-chase Axonal Long-distance (OPAL) microscopy, which utilizes well-characterized tagged channels and culture system in multi-compartment microfluidics chambers for observing molecular transport events in DRG neurons (Akin et al., 2019, 2021; Higerd-Rusli et al., 2022). We found that PTX elevated Na_V_1.8 levels at the surface of DRG distal axonal endings in a dose-dependent manner, paralleled by an accelerated delivery of Na_V_1.8-containing transport vesicles. Moreover, PTX treatment increased the apparent number of Na_V_1.8 channels per vesicle as well as increasing the number of vesicles traversing DRG axons, raising the possibility that both Na_V_1.7 and Na_V_1.8 contribute to the hyperexcitability of DRG neurons in PTX-induced CIPN.

## Methods

### Expression constructs

The codon-optimized human Na_V_1.8 plasmid (pcDNA5-hNav1.8) was previously described (Faber et al., 2012). A cassette containing the HaloTag enzyme for covalent fluorophore linkage followed by a synthetic β4 transmembrane segment was fused to the N-terminus as previously described (Higerd-Rusli et al., 2022).

### Culture of dorsal root ganglion neurons

All animal preparations adhered to protocols approved by the Institutional Animal Care and Use Committee of the Veterans Administration Connecticut Healthcare System. DRG neurons were isolated from neonatal Sprague–Dawley rats and transfected with HaloTag-Na_V_1.8 as previously described (Dib-Hajj et al., 2009; Higerd-Rusli et al., 2022). Studies of channel traffic used two microfluidic chambers separated by a physical barrier 450 μm in width with microgrooves to allow axons to extend from a soma chamber to the axon chamber (Xona Microfluidics, Research Triangle Park, NC) that were adhered to 50 mm glass-bottom dishes previously coated with poly-L-lysine and laminin as described (Akin et al., 2021). Transfected DRG neurons were applied to the somatic chambers and the growth medium (Neurobasal with 2% B27, 1% penicillin/streptomycin, 1% GlutaMAX, all from Thermo Fisher Scientific, Waltham, MA) was supplemented with 50 ng/ml NGF (Envigo, Indianapolis, IN) and GDNF (Preprotech, Windsor, NJ) the following day. The distal axonal chamber received medium with 100 ng/ml NGF and GDNF to attract growth of axons into this chamber. Cultures were treated as described below and analyzed on day 7 after preparation unless otherwise noted.

### Pharmacological treatments

Either 24 h or 48 h before analysis, half the medium was removed from each culture dish or microfluidic chamber and replaced with fresh medium containing paclitaxel such that the final concentrations would be 25 nM or 125 nM. Control cultures received an identical volume of DMSO diluted in the same manner. Some experiments used a cocktail of inflammatory mediators applied in the same manner such that the final concentrations were: 1 μM bradykinin, 10 μM prostaglandin E2, 10 μM histamine, 10 μM 5-hydroxytryptamine, and 15 μM ATP (Hockley et al., 2014).

### Labeling channels with covalently-linked fluorophores

Each microfluidic chamber of a DRG culture was perfused with prewarmed normal imaging saline (recipe) for 3 min at a rate of 0.8 ml/min with a peristaltic pump. To visualize Na_V_ channels present at the surface of neuronal endings, the distal axon chamber was perfused with 1 ml of normal imaging saline containing 100 nM of cell-impermeant Janelia Fluor 635 (JF635i) and incubated for 15 min at 37°C. For analyzing the anterograde transport of channels, the somatic chamber was perfused and incubated at the same time with 1 ml of cell-permeable Janelia Fluor 549 (JF549). Both fluorophores were a gift of Luke Lavis and Jonathan B. Grimm, Janelia Farm Research Campus, Alexandria, VA (Grimm et al., 2015; Jonker et al., 2019) and are synthetic ligands that become covalently linked to the HaloTag enzyme fused to the extracellular terminus of the Na_V_1.8 expression construct. Following incubation, each chamber was again washed with normal imaging saline for 3 min before confocal microscopy.

### Imaging of fluorophore-tagged channels

Axonal endings were identified by examining distal chambers of microfluidic dishes on an inverted Nikon Ti-Eclipse microscope (Nikon Instruments, Melville, NY) equipped with an incubation enclosure to maintain cultures at 37°C, and a Dragonfly spinning confocal disk system (Andor, Concord, MA) incorporating 561 nm and 635 nm diode lasers for high-speed imaging of living cells with limited phototoxicity. To quantify cell-surface channel expression, 0.2 μm optical sections of the axial extent of the endings were collected through a 60×/1.4 NA oil-immersion objective Nikon and a 700 nm ± 50 nm band-pass emission filter. The average JF635i fluorescence intensity of the distal 60 μm of an axon across the summed z-stack was used as the surface expression value, after subtracting the value obtained from an identically shaped region of interest in the adjacent background.

### Analysis of anterograde transport

The same axonal endings measured for cell-surface Na_V_1.8 levels were also analyzed for anterograde transport of Na_V_1.8-containing vesicles that had been labeled with JF549 applied to the somatic chamber ~20 min prior. Images were acquired on the Dragonfly spinning disk system mentioned above using the same 60× objective and a 600 nm ± 50 nm band-pass filter. For each axon, 200 images at 100 ms exposure time and 2-frame averaging were acquired with an iXon888 EM-CCD camera (Andor). Images were processed by subtracting the average fluorescence intensity of a background region of interest in each image from the entire image in ImageJ (NIH), and individual axons with moving vesicles were identified. The ImageJ plugin KymographClear (Mangeol et al., 2016) was used to generate kymographs of the fluorescence intensity of each pixel along the axon versus time in milliseconds. These kymographs were then passed to KymoButler (Jakobs et al., 2019), an algorithm developed for automated extraction of the trajectories for particles at least 3 pixels in diameter from kymographs. For these presumptive labeled transport vesicles, Kymobutler calculates the average velocity, measures the fluorescence, and identifies pauses. All analyses included only those vesicles with net anterograde motion of at least 0.1 μm/s. Summary statistics, significance testing, and visualizations of these datasets were conducted by custom routines written in Python 3.8, which are available online at https://github.com/ycnrr.

### Electrophysiology

For electrophysiological studies, neonatal rat DRG neurons were plated into coverslips coated with poly-L-lysine and laminin in medium supplemented with 50 ng/ml NGF and GDNF. Cells were incubated with 25 nM Paclitaxel or an equivalent volume of DMSO for control. Following 24 h of treatment, coverslips were taken for voltage-clamp recordings.

Patch pipettes were fabricated from borosilicate glass (World Precision Instruments, Sarasota, FL) using a P-97 puller (Sutter Instruments, Novato, CA) and fire-polished for a resistance of 0.8–1.2 megaohms when filled with internal solution. The pipette internal solution contained (in mM): 140 CsF, 10 NaCl, 1.1 EGTA, 10 HEPES, 20 Dextrose (pH 7.3 with CsOH, adjusted to 310 mOsm/l with dextrose). External bath solution contained (in mM): 140 NaCl, 20 TEA-Cl, 3 KCl, 1 CaCl_2_, 1 MgCl_2_, 10 HEPES, 5 Sucrose, 0.1 CdCl_2_, 0.001 TTX (pH 7.3 with NaOH, adjusted to 320 mOsm/l with sucrose).

DRG neurons between 25 and 30 μM were selected due to the relatively high expression of Na_V_1.8 in neurons of this size (Shields et al., 2012). Macroscopic currents were recorded in voltage-clamp mode using an EPC-10 amplifier and the PatchMaster Next program (HEKA Electronik, Lambrecht, Germany). Sodium currents were recorded in the whole-cell configuration. Cells with a leak current >200 pA were excluded. Series resistance compensation of 80–90% was applied to reduce voltage error. Cells were excluded if the voltage error exceeded 5 mV. Recordings were sampled at 50 kHz through a low-pass Bessel filter of 10 kHz. After achieving the whole-cell configuration, a 5-min delay was applied to allow adequate time for the pipette solution and cytoplasmic milieu to equilibrate.

As PTX-treated neurons were maintained in culture for over 48 h, extensive neurite outgrowth caused inadequate space clamp. To isolate somatic sodium currents from inadequately clamped axonal currents, a voltage pre-pulse protocol, as described by Milescu et al. (2010) was implemented. Briefly, neurons were held at a potential of −80 mV (to inactivate non-Na_V_1.8 TTX-R channels). Then, patched cells were pre-pulsed to an individualized potential for 8 ms to inactivated axonal sodium currents. This prepulse potential was found to be the most negative voltage that could trigger an axonal spike without activating somatic channels – usually −35 to −25 mV. This was then followed by returning the cells to a hyperpolarized interpulse potential for 1 ms to allow for somatic but not axonal channel recovery. The interpulse potential was determined as the most negative potential that would not recover inadequately clamped sodium channels – usually between −100 and −120 mV. Finally, somatic sodium currents were triggered by subsequent 100 ms test pulses from −80 to +40 mV in 5 mV increments.

I-V relationships generated by the voltage protocol were fit according to the following equation:


$$I=\frac{G_{\max}\left(V-V_{rev}\right)}{\left(1+e^{\frac{\left(V_{0.5}-V\right)}{k_{G}}}\right)}$$

Where I is the peak current for the test potential V, V_rev_ is the reversal potential, G_max_ is the maximum channel conductance, V_1/2_ is the half-maximal activation potential, and k_G_ is the slope factor.

### Real-time PCR of DRG neurons

DRG neurons were cultured and treated with either 25 nM PTX or an equivalent volume of DMSO for 24 h as done for voltage-clamp recordings (above). Total RNA was then isolated according to the directions in the RNeasy Plus Mini Kit (Qiagen, Germantown, MD). Complementary DNA was produced using iScript™ Reverse Transcription Supermix (Bio-Rad, Hercules, CA), and one tenth of each reaction was used in 20 μl Bio-Rad PrimePCR™ probe assays for beta actin (qRnoCIP0050804) or Scn10a (qRnoCIP0027343) with SsoAdvanced supermix on the Bio-Rad CFX96 Touch System. Thermal cycling parameters included a 30s initial denaturation step at 95°C, followed by 40 cycles of 15 s at 95°C and 30s at 60°C. RNA quality was verified with the PrimePCR™ RNA Quality Probe Assay. Three independent dishes were processed in parallel for each treatment condition. Relative expression levels were derived from the ΔΔC_t_ method and normalizing to beta actin using Bio-Rad CFX Manager software.

## Results

### Paclitaxel treatment increases levels of Na_V_1.8 channels at the surface of DRG axon endings

**Figure 1:**
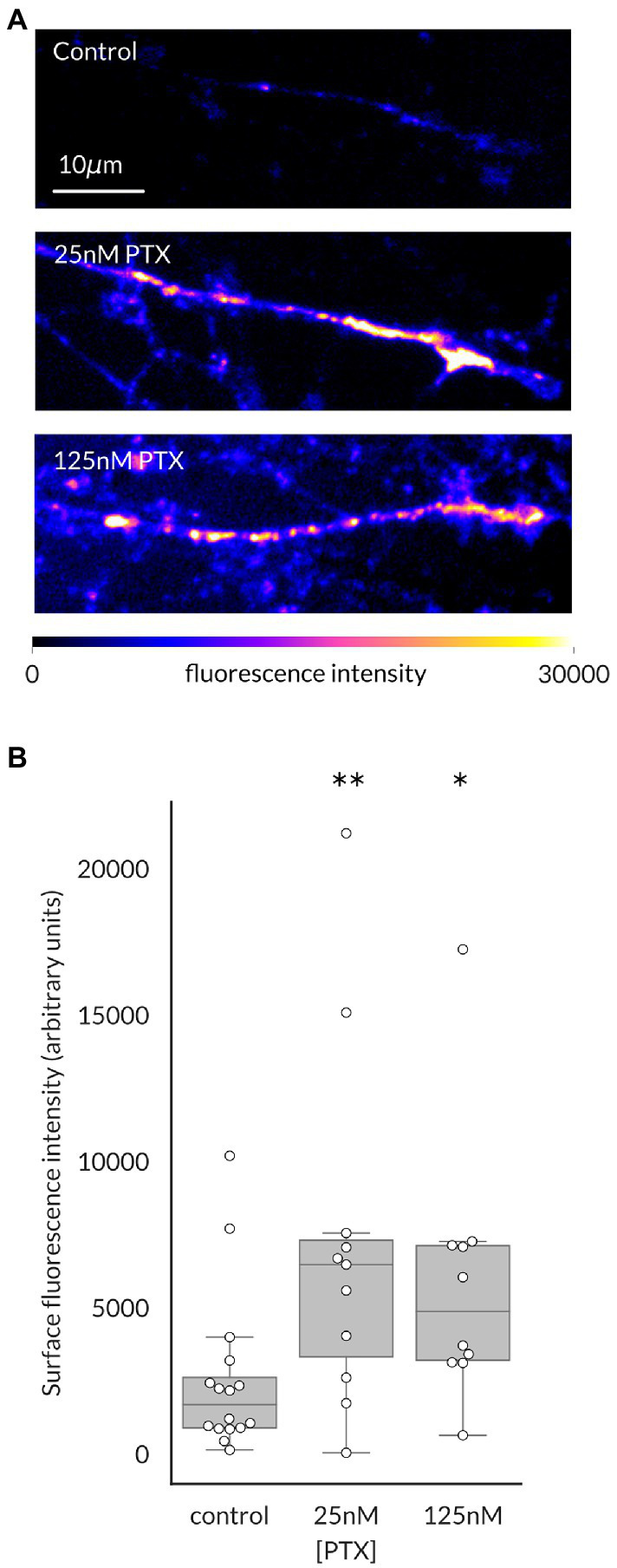
Paclitaxel treatment increases Na_V_1.8 channels at the surface of distal axons of DRG neurons. **(A)** Representative maximum intensity projections from confocal stacks of DRG axonal endings in the distal portion of the microfluidic chamber, pseudocolored by fluorescence intensity of labeled Na_V_1.8. **(B)** 24 h of treatment with 25 nM or 125 nM PTX increased surface level of Na_V_1.8 at axonal endings (**p = 0.009 and *p = 0.011 respectively, by Bonferroni-corrected Mann–Whitney U test). At least 3 independent cultures were analyzed per condition; each point indicates an individual axonal ending (N = 16, 11, and 10 axons, respectively, for each treatment group).

We have previously shown that surface level of Na_V_1.7 channels at the endings of DRG axons in culture is elevated by paclitaxel (PTX) treatment, which was paralleled by increased transport of anterogradely moving vesicles containing higher numbers of channels (Akin et al., 2021). We first used this methodology to determine if this was also true for Na_V_1.8 channels, as suggested by its cotransport with Na_V_1.7 channels (Higerd-Rusli et al., 2022). Indeed, increased levels of surface level of Na_V_1.8 in distal axonal ends of DRG neurons were apparent after 24 h of treatment with either 25 nM or 125 nM PTX (Figure 1).

### Enhanced anterograde transport of Na_V_1.8 channels

**Figure 2:**
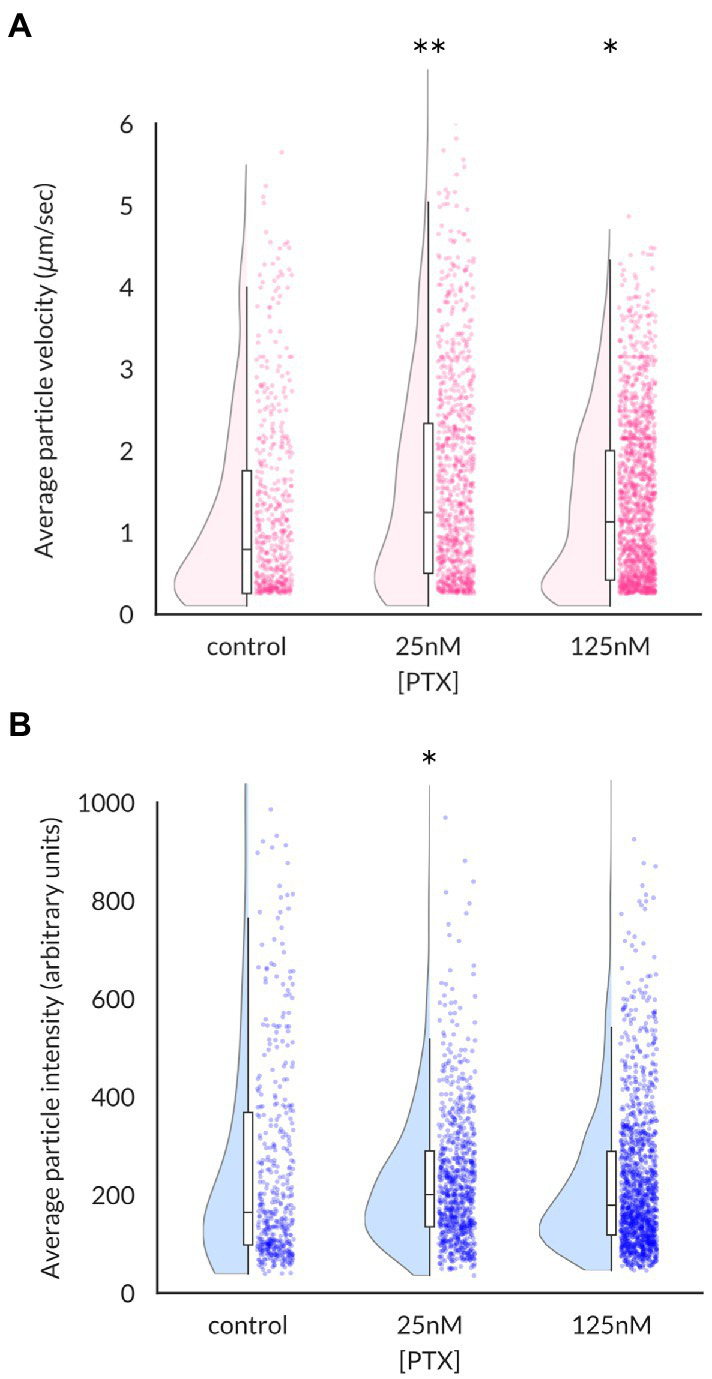
Effects of paclitaxel on content and velocity of vesicles carrying Na_V_1.8 channels to distal axons. **(A)** Violin plots and box plots showing treatment of DRG neurons for 24 h with 25 nM or 125 nM PTX enhances the average velocity of Na_V_1.8-containing particles (**p = 6.3 × 10^−8^ and *p = 4.5 × 10^−6^ by Mann–Whitney U test with Bonferroni correction, respectively). Each point indicates the average velocity of a particle over the 40s imaging epoch; at least 3 independent DRG cultures were assayed for each condition. **(B)** Median vesicular fluorescence intensity was significantly increased by PTX treatment of 25 nM (*p = 0.02 by corrected Mann–Whitney U test).

Increased levels of Na_V_1.8 channels at the surface of axonal endings could arise from increased stability of channels at the plasma membrane or enhanced delivery of channels. The microfluidic chamber culture system provides an ideal method to distinguish between these possibilities by monitoring the traffic of channel-containing vesicles as they migrate in real time. The average velocity of Na_V_1.8-containing vesicles, defined as the distance traveled over the duration of the imaging epoch, was significantly enhanced by PTX treatment (median (95% confidence interval): 0.79 (0.68–0.89) μm/s for control, 1.25 (1.12–1.40) for 25 nM, and 1.13 (1.04–1.22) for 125 nM; Figure 2A). The median vesicle fluorescence intensity at the 25 nM dose was greater than that of control cultures, suggesting increased loading of Na_V_1.8 channels into vesicles (median (95% confidence interval): 164.7 (147.1–183.4) for control, 200.6 (190.5–210.1) for 25 nM PTX, and 179.0 (172.5–189.1) for 125 nM PTX; Figure 2B).

**Figure 3:**
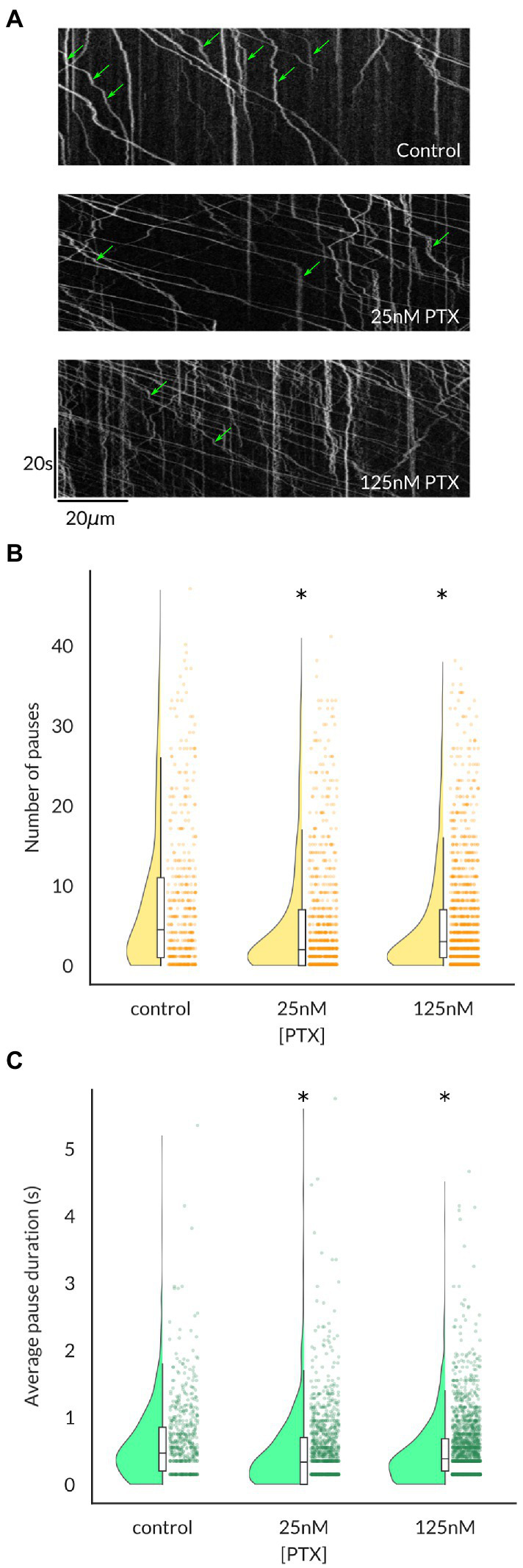
Vesicles containing Na_V_1.8 pause less frequently and for shorter durations following paclitaxel treatment of DRG neurons. **(A)** Kymographs show the position of fluorescent vesicles along the trajectory of an axon (x-axis) as a function of time (y-axis). Stationary periods (green arrows) appear as vertical lines, which decrease both in frequency **(B)** and average duration **(C)** under PTX treatment. Each point in **(B**,**C)** represents an individual vesicle and at least 3 independent cultures were analyzed per condition (* all *p <* 7.6 × 10^−5^ by Mann–Whitney U test corrected for multiple comparisons).

**Figure 4:**
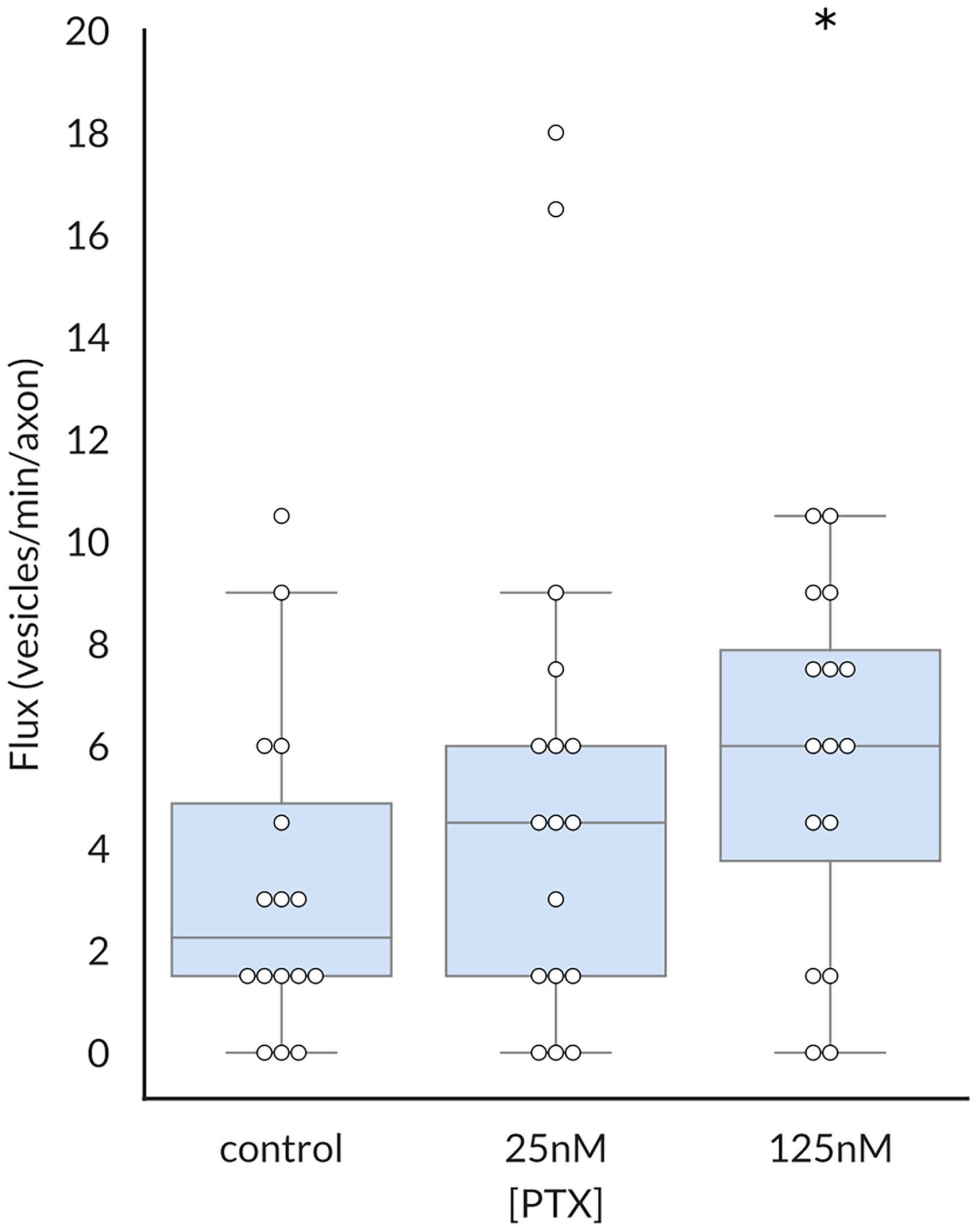
Paclitaxel enhances flux of vesicles containing Na_V_1.8. The vesicular flux, defined as the number of vesicles traversing half of the imaged axon per minute, was significantly increased by treatment with 125 nM PTX (*p* = 0.025 by corrected Mann–Whitney U test). At least 3 independent cultures were analyzed per condition; each point indicates an individual axon.

Because the average vesicular velocity measured over the entire imaging period does not reflect any irregular changes in vesicular movement, we also recorded the frequency and duration of vesicle pauses, both of which were decreased by PTX (Figure 3). Moreover, there were more vesicles detected under PTX treatment conditions in general. The number of vesicles traversing half of the imaged axon per minute, termed the vesicular flux, was significantly increased at the 125 nM dose (Figure 4). Therefore, PTX treatment not only increased the surface levels of Na_V_1.8 channels through increased channels per vesicle, but through increased vesicles per axon and accelerated delivery with fewer and shorter pauses along the transport route.

### Channel gene expression and current density

**Figure 5:**
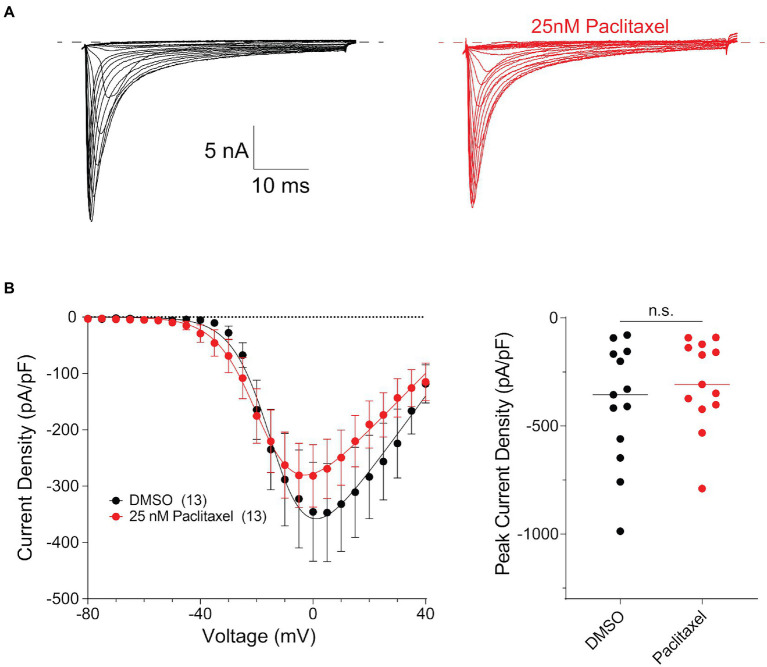
Paclitaxel does not affect density of current produced by endogenous Na_V_1.8 in the somas of DRG neurons. **(A)** Representative family of somatic Na_V_1.8 currents evoked by 100 ms depolarizing voltage steps from −80 to −40 in 10 mV increments from a holding potential of −80 mV. Traces from rat DRG neurons treated with DMSO control (black) and 25 nM PTX (red) conditions are displayed. Traces are shown only for current evoked during the test-pulse and do not show the response from the pre-pulse stimulus. **(B)** Left panel shows current density-voltage relationships of currents produced by endogenous somatic Na_V_1.8 channels in neonatal rat DRG neurons treated with DMSO (black; *n* = 13) or 25 nM PTX (red; *n* = 13). Data are presented as means ± SEM. Right panel shows distribution of peak current densities of cells. Means indicated by horizontal bars, no statistically significant differences were detected by Student’s *t*-test (*p* > 0.05).

To examine potential functional consequences of PTX treatment on Na_V_1.8 surface levels in DRG neurons, we examined the Na_V_1.8-dependent current density in DRG neuron cultures after treating cells for 24 h with 25 nM PTX. Endogenous Na_V_1.8-dependent current density was unaffected by PTX treatment (Figure 5). This is consistent with the observation that expression of endogenous Na_V_1.8-encoding mRNA by quantitative RT-PCR was 1.04 ± 0.26 (average ± s.d.) fold in PTX-treated cultures relative to 1.01 ± 0.13 in controls.

## Discussion

We show in this study that surface levels of Na_V_1.8 channels at the axonal endings of cultured DRG neurons are elevated by treatment with PTX. Vesicles carrying fluorescently labeled Na_V_1.8 channels moved with greater velocity and stopped less frequently and for shorter intervals along axons in PTX-treated neurons. Such vesicles were greater in number, and the estimated Na_V_1.8 content of each vesicle tended to be larger. Our data suggests that the greater vesicular flux and accelerated delivery of channels *via* microtubule-based transport are very likely to have contributed to the increased levels of the channel at the distal axonal surface.

These observations are consistent with our previous data demonstrating co-trafficking of Na_V_1.7 and Na_V_1.8 channels in anterograde transport vesicles (Higerd-Rusli et al., 2022), and the PTX-induced increase in trafficking of Nav1.7 in sensory neurons (Akin et al., 2021). PTX treatment increases the flux, number of channels per vesicle and the transport velocity of these vesicles, resulting in elevated surface expression of both channels at axonal endings. The flux of Nav1.8-carrying vesicles was increased by treatment with 25 nM PTX, but this increase only reached statistical significance when cultures were treated with 125 nM PTX. This contrasts with our previous observations of Na_V_1.7-carrying vesicles, which showed statistically significant increase in vesicular flux in cultures treated with 25 nM PTX (Akin et al., 2021). One explanation for this discrepancy is that because the vesicular colocalization of these two channels is not perfect (about 67% of vesicles carry both channels), it remains possible that lower doses of PTX mobilize a vesicular pool containing a greater proportion of Na_V_1.7 relative to Na_V_1.8 channels. Another difference between our two studies is that unlike the effect of PTX on Nav1.7 expression and current density at the soma, PTX treatment did not increase levels of Nav1.8 mRNA or Nav1.8 current density. This suggests that the effects of PTX on the expression of the two channels and trafficking to the soma is distinct, while the effects on axonal trafficking of the channels and accumulation at the surface of axonal ends are similar.

Choosing appropriate PTX concentrations to model the *in vivo* pharmacokinetics of clinical treatment remains a challenge. Plasma PTX levels in human patients reach peaks of over 1 μM (Ohtsu et al., 1995) but decline to ~20 nM in the 48 h after infusion (Andersen et al., 2006). Animal studies suggest that PTX can accumulate to high levels within tissues and persist for a week or more (Xiao et al., 2011). In some DRG neuron culture systems, high nanomolar concentrations of PTX cause formation of retraction bulbs suggestive of axonal degeneration, particularly with extended exposure (Akin et al., 2021). Some DRG cultures have been shown to survive even higher concentrations of PTX with only 24 h of treatment, however, exhibiting increased excitability and spontaneous activity after 48 h of recovery relative to cells that were never exposed to PTX (Villalba-Riquelme et al., 2022). In the current study we therefore focused on only 24 h of exposure to a modest dose (25 nM) of PTX that has previously been associated with alterations in ion channel transport and a moderate dose (125 nM) below that previously associated with axonal retraction (Akin et al., 2021). This moderate dose still showed reduced vesicular velocity and Na_V_1.8 content relative to the lower dose while remaining greater than that in the control cultures, raising the possibility that increasing PTX doses begin to induce some toxicity that could select for sampling of subtypes of unmyelinated sensory neurons.

What are the functional consequences of increased Na_V_1.7 and 1.8 channel levels at the surface of DRG axonal endings? Na_V_1.7 sets the threshold for action potential firing (Vasylyev et al., 2014; Alexandrou et al., 2016), whereas Na_V_1.8 contributes most of the current under the depolarizing phase of the action potential, regulates spike width and contributes to the ability to sustain repetitive discharge (Renganathan et al., 2001; Blair and Bean, 2002; Han et al., 2015). Presumably increased levels of these channels at the surface of endings of axons, where action potentials are initiated in DRG neurons, would render them more likely to generate action potentials and sustained bursts. Indeed, PTX treatment increases spontaneous activity of nociceptors *in vitro* (Villalba-Riquelme et al., 2022) and *in vivo* (Li et al., 2018), and increases the proportion of cultured DRG neurons that exhibit sustained firing upon current injection (Villalba-Riquelme et al., 2022). Moreover, Villalba-Riquelme et al. also reported that PTX effects varied between isolectin B4-postive and negative subtypes of nociceptors, raising the possibility that neuronal heterogeneity could contribute to variability between different experiments. It is noteworthy that there are relatively wide ranges of vesicular velocities and channel expression levels for individual axons in our culture system, and this system does not currently allow correlation of these measurements with the electrophysiological properties of the same cells measured at the soma. It is possible that the increased neuronal activity of DRG neurons after treatment with PTX might have been driven primarily by the increased levels of Nav1.7 because we did not see a treatment-induced increase in the Nav1.8 current density at the soma.

Together with our own previous results and those of other investigators, our current findings emphasize the importance of subcellular localization of ion channels in sensory neurons. Manipulations without a significant effect on bulk channel expression at the mRNA or protein level could nevertheless alter the localization of channels and thus influence propagation of electrical signals. Because there are separate vesicular pools for different molecules moving to axonal endings, regulation of vesicular transport remains a possible therapeutic target for processes dependent on ion channel distribution. However, co-trafficking of multiple types of channels within the same vesicles suggests that manipulations of specific subsets of transport vesicles do not just affect one channel in isolation, but all molecules within that vesicular pool. Understanding the process by which molecules are sorted into these pools, and how to alter delivery of those vesicles to their destinations, will enable greater therapeutic efficacy against not only chemotherapy-induced peripheral neuropathy but other painful sensory disorders.

## Data availability statement

The raw data supporting the conclusions of this article will be made available by the authors, without undue reservation.

## Ethics statement

The animal study was reviewed and approved by Institutional Animal Care and Use Committee of the Veterans Administration Connecticut Healthcare System.

## Author contributions

CAB conducted experiments on surface channels, vesicle traffic, and mRNA expression, analyzed the data and wrote the manuscript. ST conducted electrophysiology experiments, analyzed data, and wrote the manuscript. GPH-R conducted pilot experiments and provided suggestions. SL and PZ established culture systems for the examination of channel transport and current density in DRG neurons. FBD-H engineered DNA constructs for labeling sodium channels in transfected cells. SGW and SDD-H conceived the project, provided suggestions, and reviewed the manuscript. All authors contributed to the article and approved the submitted version.

## Funding

This work was funded by U.S. Department of Veterans Affairs Rehabilitation Research and Development Service awards RX003621 and RX002999 and Biomedical Laboratory Research and Development Service BX004899 to SGW and SDD-H. GPH-R is supported by NINDS NS122417-01. GPH-R and ST are supported by NIH/NIGMS Medical Scientist Training Program GM007205. The Center for Neuroscience and Regeneration Research is a Collaboration of the Paralyzed Veterans of America with Yale University.

## Conflict of interest

The authors declare that the research was conducted in the absence of any commercial or financial relationships that could be construed as a potential conflict of interest.

## Publisher’s note

All claims expressed in this article are solely those of the authors and do not necessarily represent those of their affiliated organizations, or those of the publisher, the editors and the reviewers. Any product that may be evaluated in this article, or claim that may be made by its manufacturer, is not guaranteed or endorsed by the publisher.
